# Healthcare providers’ experience of identifying and caring for women subjected to sex trafficking: a qualitative study

**DOI:** 10.1186/s12905-024-02992-6

**Published:** 2024-02-29

**Authors:** Mikaela Andersson, Karin Örmon

**Affiliations:** 1 School of Public Health and Community Medicine, Institute of Medicine, University of Gothenburg, Gothenburg, Sweden; 2 The Västra Götaland Region Competence Centre on Intimate Partner Violence, Gothenburg, Sweden; 3 Department of Health, Blekinge Institute of Technology, Valhallavägen 1, Karlskrona, 371 41 Sweden

**Keywords:** Global health, Healthcare providers, Human trafficking for sexual exploitation, Qualitative research, Women’s healthcare

## Abstract

**Background:**

Men’s violence against women, including human trafficking for sexual exploitation, is a severe threat to global health. Healthcare providers are uniquely positioned to identify and care for women subjected to human trafficking for sexual exploitation. They are among the few professionals the women interact with while being exposed to human trafficking for sexual exploitation. This study aims to describe healthcare workers’ experience of identifying and caring for women subjected to human trafficking for sexual exploitation seeking women’s healthcare.

**Method:**

A qualitative design was chosen and nine qualitative interviews with healthcare providers were conducted and analyzed using the content analysis method.

**Results:**

Three main categories were revealed: (1) the importance of being attentive, (2) the importance of providing safety, and (3) the importance of collaborating, followed by a number of subcategories: behavioral and physical signs, limited time to interact, security measures, value of confidence building, organizational collaboration, essential external network, and information transmission.

**Conclusions:**

As the women subjected to sex trafficking have limited time in healthcare, it is important for healthcare providers to be attentive and act immediately if suspecting human trafficking for sexual exploitation. It may be the only possibility for the healthcare providers to care for these women and reach them. They must endeavor to provide the women with safety due to their vulnerable position at the hospital. However, these women may leave the healthcare setting unidentified and unaided, which highlights the importance of collaboration on multiple levels.

## Background

Violence affects about one-third of women globally, and the perception of violence against women has changed during the past decades [1]. What was once seen as a private concern is now considered a severe global issue that violates human rights and endangers world health [1]. The Istanbul Convention is the first legally binding declaration about violence against women [2]. It aims to reduce all violence against women and all domestic violence [2]. Human trafficking for sexual exploitation is part of the umbrella concept of violence against women [3]. The UN definition of human trafficking is stated in the Palermo Protocol, which most countries agreed upon in 2000 [3]. The Palermo Protocol describes human trafficking as follows:

… the recruitment, transportation, transfer, harboring or receipt of persons, by means of the threat or use of force or other forms of coercion, of abduction, of fraud, of deception, of the abuse of power or of a position of vulnerability or of the giving or receiving of payments or benefits to achieve the consent of a person having control over another person, for the purpose of exploitation… [3, p.3].

In Sweden, the definition of men’s violence against women includes both human trafficking for sexual exploitation and all forms of prostitution [4]. It is however, legal to sell sex within the country, but not to buy sexual services [5]. An individual only receives formal status as a victim of human trafficking if the individual is a plaintiff in a preliminary investigation [6].

The European Charter for Equality of Women and Men in Local Life, that is, a declaration promoting gender equality at local and regional level, is another declaration involving human trafficking [7]. The aim of the declaration is to encourage municipalities and regions within Europe to work together to improve the equality among the residents [7]. Overall, signing the declaration means that human trafficking must be treated as a violation of human rights and each party must act preventively against human trafficking [7]. Preventive measures that can be taken are, for example, training programs for specialist teams [7]. For the healthcare providers, signing the declaration means delivering the highest possible level of care [7]. In addition, there must be effective collaboration between the various authorities, such as the healthcare and the police [7]. The region of Västra Götaland in Sweden adopted the declaration in 2008 [8].

Although human trafficking for sexual exploitation has existed for a long time, it has become more frequent due to neoliberal globalization [9]. Economic conditions, accessibility of travel, and less official oversight, have driven more poor individuals into the exploitation of sex trafficking [9]. Trade-opening countries, on the other hand, may have less forced labor and enhanced economic rights for women and thus a reduced risk of people being subjected to sex trafficking [9]. It is challenging to measure the precise number of individuals being subjected to human trafficking for sexual exploitation, because the exploitation is illegal [10]. However, the 2021 Global Estimates indicate that 6.3 million are subjected to forces of commercial sexual exploitation [11]. The majority are girls and women [11]. In 2022, the Swedish authorities suspected 223 women of being subjected to human trafficking and human exploitation, primarily human trafficking for sexual exploitation [12]. The women were predominantly from Romania, Ukraine, Thailand, Bulgaria, and Nigeria [12].

Commonly, women are recruited into human trafficking for sexual exploitation by an intimate partner, family member, or other relative [13]. The average duration of women’s subjection to human trafficking for sexual exploitation is 1.8 years [13]. The crime, and the severe violation of human rights, will remain as long as there is a demand for buying sexual services [14].

### The healthcare setting

Healthcare providers have a unique and essential position in identifying and caring for patients subjected to human trafficking for sexual exploitation [15]. The healthcare providers are among the few professionals interacting with women subjected to human trafficking for sexual exploitation [16]. They can offer essential medical and psychological care, as the women’s experiences and circumstances put them at risk of a variety of health problems [17]. However, research on healthcare providers’ role in decreasing sex trafficking is limited [18]. Previous research on human trafficking for sexual exploitation demonstrates several challenges in accessing healthcare, both during and after escaping the exposure [19]. Obstacles may include navigating the healthcare system, making an appointment, and getting to the healthcare facility [20]. The trafficker attendance in the hospital also challenges the possibility of private conversations between the women and healthcare providers [19]. Despite barriers, most women subjected to sex trafficking receive healthcare at some point during the exploitation [21]. Previous research shows that 87.8% of 98 women subjected to sex trafficking obtained healthcare [21]. Women’s healthcare is one of the most common settings for these women to seek care [22]. Research on the health implications of human trafficking for sexual exploitation demonstrates long-lasting health effects [23]. Primarily, outcomes on physical health, such as acute physical injuries, gynecological conditions, and infectious diseases, and on mental health [24]. Due to limited access to healthcare the women often have acute and severe medical conditions when seeing a healthcare professional [24].

Although the women in question seek medical care, patients subjected to human trafficking for sexual exploitation may leave the hospital unrecognized and unaided [21]. Healthcare providers have stated challenges in identifying patients subjected to human trafficking for sexual exploitation, e.g., lack of knowledge and language barriers [18]. Moreover, persons subjected to human trafficking for sexual exploitation have emphasized that the healthcare providers’ lack of knowledge, awareness, and empathy creates shame and fear in the patients in question when visiting healthcare [25]. Hence, the Regional Council have decided that all healthcare providers in the Västra Götaland region, must receive training regarding intimate partner violence [26]. Furthermore, all healthcare professionals who make healthcare assessments must obtain knowledge regarding routine inquiry about violence in patient meetings [26]. Knowledge about human trafficking for sexual purposes has been included in this assignment [26]. Additionally, the healthcare professionals must provide acute care to the patient regardless of the purpose of the person’s stay in Sweden [27]. To a certain extent, asylum seekers in Sweden get acute care for free [27]. Asylum seekers under the age of 18 have the same rights to care as other children in Sweden, the care being for the main part free of charge [27].

Research on healthcare providers’ role in decreasing sex trafficking is narrow [18] and empirical research on human trafficking for sexual exploitation is lacking [28]. Existing literature is mainly found within the grey literature [28]. There are also knowledge gaps regarding human trafficking for sexual exploitation, since human trafficking is often discussed as a combined concept rather than broken down into trafficking forms [28]. Therefore, the aim of this study is to describe healthcare workers’ experience of identifying and caring for women subjected to human trafficking for sexual exploitation seeking women’s healthcare.

## Methods

We have chosen a qualitative design for the study, which means that the study is based on examining the meaning individuals attribute to situations [29]. A qualitative design aims to illuminate individuals’ subjective experiences [30]. Content analysis by Burnard [31] was chosen for a structured manifest analysis of the interviews.

### Study participants and setting

This qualitative interview study applied a purposive sampling aiming to collect respondents that could provide deep and essential information about the topic [29]. The inclusion criteria were to work as a healthcare provider within women’s healthcare and to have experience of identifying and caring for a woman subjected to human trafficking for sexual exploitation. Relevant experiences included the healthcare providers’ suspicions of human trafficking for sexual exploitation. The exclusion criteria were healthcare providers with no direct patient contact.

Accessing the healthcare workers required multiple permissions. The initial step was to contact the women’s healthcare operations manager for confirmation, and the operations manager was emailed with attached information about the study. The operations manager gave permission to perform the research and attached contact information to the four required section managers. All the section managers accepted the request and gave contact information to the health unit managers. One of the researchers then contacted all the healthcare unit managers and the same attached information about the study as the information to previous authorities. Four healthcare unit managers invited the researcher to meet the healthcare workers to provide information about the research and answer potential questions. An information letter was distributed to the healthcare workers who attended the meetings. The rest of the healthcare workers were given the same information by email.

Some healthcare workers expressed interest during the meetings and explicitly offered to participate, while others expressed interest via email. Moreover, all respondents were asked if they knew anyone in women’s healthcare who they thought might be interested in participating in the study. That resulted in identifying another two healthcare providers, so-called snowball sampling [30]. Finally, nine healthcare providers, representing six healthcare units, signed up for interviews. None of the healthcare providers refused to participate in the study or withdrew their participation. See Table 1 for socio-demographic data of participants.

**Table 1:** Socio-demographic data of participants (n = 9)

**Gender** Female Male	9 0
**Age** 25–35 36–45 46–55 56–65	2 2 2 3
**Profession** Doctor Midwife Nurse Assistant nurse	1 6 1 1
**Working experience** 1–10 years 11–20 years 21–30 years 31–40 years	4 1 3 1

### Data collection

The interviews were conducted in six healthcare units within women’s healthcare in a larger city in western Sweden: the Department of Abortion and Gynecology, the Department of Gynecology, the Gynecology Clinic, the Gynecological Emergency Department, the Specialist Delivery Department, and the Specialist Maternal Care. Initially, the goal was to involve all departments at women’s healthcare equally; however, the Department of Abortion and Gynecology is the most represented department in the study.

An interview guide was created, with open-ended questions. The interview guide included six introductory questions, followed by five detailed questions focused on the research aim. The questions in the interview guide encouraged the healthcare workers to speak freely about the issue. In order to understand what type of answers the questions would generate, the flow, and the approximate time needed to complete the interview, the interview process began with a pilot interview [32]. The healthcare workers did not have any objections to the interview guide, and the researchers found that the questions addressed the research aim. Therefore the interview guide remained unchanged.

The duration of the interviews was between 40 and 70 min, with an average of 55 min. They were conducted in Swedish, recorded, and transcribed, and later translated into English by a professional translator, well experienced in the English language. The quotes have been translated verbatim. All interviews were performed by the first author (MA) and collected between February and April 2023.

### Data analysis

The interview material was analyzed in accordance with Burnard’s [31] manifest content analysis, described in fourteen stages. The aim of the content analysis is to create a thorough and organized record of the topics and problems raised in the interviews and connect the themes and interviews in a logical categorization scheme. The first steps, 1–3, aim at immersion in the data by making notes and memos of the transcribed material and doing the open coding. The next steps, 4–7, focus on collapsing categories that are similar into broader categories and then creating a list of new categories and sub-headings. The category system was reviewed along with the transcripts, and it was reduced into broader themes [31].

Steps 8–10 focus on working through the list of categories and sub-headings in the transcripts using colored highlighting pens. Coded sections of the transcripts were cut out and put onto sheets, organized with headings and sub-headings. Step 11 involves the respondents controlling the system; however, that step was not included since the analysis is discussed from a group perspective rather than a single-person perspective. The final steps, 12–14, focus on the writing [31].

### Rigor

The authors aimed for rigor, in accordance with standard criteria for qualitative research [33]. To ensure dependability, the participants were given the same core questions, structured in the interview guide. Burnard’s manifest content analysis [31] assisted in developing the most appropriate categories for the research and in excluding irrelevant data. The results are clearly stated, and quotations from the transcripts are used in the analysis to strengthen the choice of themes. To control the researchers’ preunderstanding of women’s healthcare and human trafficking for sexual exploitation, the analyzing process was slowed down, aiming to be open to the findings. Time was taken to reflect on the coding of the material and listen to the recorded interviews several times in order to understand what the healthcare providers indicated. By being conscious of their preconceptions, the researchers attempted to lessen the impact of those preconceptions on the results and achieve openness.

## Results

The findings regarding the healthcare providers’ experiences of identifying and caring for a woman subjected to human trafficking for sexual exploitation are organized under three headings: the importance of being attentive, the importance of providing safety, and the importance of collaborating, followed by sub-headings in accordance with Burnard [31]. See Table 2 for headings and sub-headings.

**Table 2:** The sub-categories and categories of the result, with meaning units

Meaning Units	Subcategories	Categories
Body injuries or certain actions within the hospital can be signs of sex trafficking. Acting swiftly is essential since the women have limited time within the hospital.	Behavioral and physical signs Limited time to interact	**The importance of being attentive**
Tools are given to identify and care for the females. The hospital should be an accepting place, in order to enable identifying the women.	Security measures Value of confidence building	**The importance of providing safety**
The healthcare providers need to be on the same path and gather around the women. The police, social services and women’s emergency services are also essential in the work. Information should be exchanged with other actors as well as communicating with the female.	Organizational collaboration Essential external network Information transmission	**The importance of collaborating**

### The importance of being attentive

The findings underline the importance of being attentive to behavioral and physical signs in order to be able to identify and care for women subjected to human trafficking for sexual exploitation. Moreover, the patient population is described as having limited time within women’s healthcare, which emphasizes the importance of acting swiftly.

### Behavioral and physical signs

According to the informants’ narratives, patients who give an impression of fear when they enter the hospital, exhibit behavioral signs that make the healthcare providers suspect human trafficking for sexual exploitation. The fear can be illustrated by the patient sitting huddled on the examination bed or seeming distracted. Behavioral acts pointing to the woman not wanting the offered care, and to the accompanying person having pushed for the care, have given the health care providers the impression that abortions were in some cases a procedure that was forced on the woman. Furthermore, avoiding questions, presenting disjointed stories, and/or getting upset or angry with the healthcare professionals, are aspects that the narratives highlight as behavioral indicators.

Redness and soreness in the female genital area are common after sexual exposure and something that it is essential to be attentive to. Depending on what the women have been exposed to, a vaginal rupture can occur, resulting in extensive bleeding. If items have been used inside the vagina or the anal area, bleeding from the vagina or anal fissures can ensue. However, injuries in the genital area are rare and mostly heal swiftly. If the women seek care the same day or within a few days of the sexual exposure, the medical signs can be identified. Permanent genital injuries are described as uncommon, but soreness in the genital area is more common among the women seeking care, which could indicate hard or persistent penetration.

*Mainly, there is a soreness if the doctor feels inside of the vagina, it is very sore, or in the anal opening, depending on what has happened, it is mainly a soreness. Injuries are very rarely seen*. (Informant 9)

Additionally, abdominal pain due to advanced pregnancies and sexually transmitted diseases are described as medical signs to be attentive to. Moreover, the abuse can be visible outside, on women’s bodies, including bruises and marks from, e.g., punches and kicks. Aside from physical signs, being attentive to the woman’s psychological well-being is important. Being subjected to sexual exploitation involves a substantial risk of long-term impacts on the women’s lives and enhanced suffering. The women were in some cases not aware of the medical risks they incurred, which points to the need for healthcare workers to perform a thorough examination.

*She felt sick and sought care, she realized that she was pregnant, and at the first medical check-up, she was confirmed to have both HIV and hepatitis and was also pregnant. Then it was revealed that she was subjected to human trafficking.* (Informant 2)

#### Limited time to interact

The narratives highlight the importance of being attentive and responding immediately to the women, as women trafficked for sexual exploitation have limited time at the hospital and limited interaction with maternal healthcare during pregnancy. The women are sometimes accompanied by a male person, who demands a quick discharge. This implies limited time to interact with the women. The limited time challenges the possibility of forming an idea of the women’s living situation as well as the ability to help and protect the women.

*It happens that they get injured by a violent customer, and they bleed and stuff, then they can’t work, then they have to come here, and then the men usually accompany them, saying “fix,” so that she can come back*. (Informant 4)

Besides the challenges of identifying the woman due to the limited time she spends in the healthcare unit, the caring can be hampered. A described challenge is to find tools to keep the woman within the healthcare, especially in pressured settings like having the trafficker strolling outside of the hospital. Long waits before seeing a doctor may cause the women to leave the hospital and maybe not return. The woman may be in another city or country the next day, which stresses the importance of being attentive, acting quickly, and investigating the reasons behind the limited time the woman stays in the clinic. However, setting aside time, prioritizing based on limited information, and listening to what the women indicate can be challenging in healthcare.

*She disappeared swiftly after leaving the hospital… I tried to reach her, but she was already out of the country*. (Informant 9)

### The importance of providing safety

The results indicate the importance of providing safety through security measures and by building confidence. Several aspects emphasize the importance of providing women with safety and also the importance of mechanisms to identify and care for a woman subjected to human trafficking for sexual exploitation. Providing the women with safety involves creating a secure place within the hospital, i.e., a place and a time when they are not monitored by the trafficker. Safety is also about giving the woman the care she needs and requires.

#### Security measures

Security measures increase the likelihood of identifying if the woman is subjected to human trafficking for sexual exploitation and of providing safety as well as giving the necessary care. A security measure aiming to offer both the women and the healthcare providers safety is the documentation in the medical charts. Documentation can be locked so that the perpetrator will not have access to the woman’s medical charts, and the executor caring for the woman can be anonymous after making notes in the medical chart.

*Some desire to have the entire medical file locked since there is someone standing behind them and watching… forcing them to log in so they can read the medical file.* (Informant 7)

Another security measure when suspecting human trafficking for sexual exploitation, is to offer the women to stay in the hospital for non-medical reasons. That creates a safe place for the women, and it is possible to provide additional care. A described obstacle to this is if the trafficker understands that the women are being hospitalized for non-medical reasons and stops transporting them to the women’s healthcare, which would enhance the risk for women subjected to sex trafficking and endanger their health.

*We have hospitalized patients who do not need to be hospitalized for medical reasons. We have told the men that, I am sorry, she cannot come, she has to be hospitalized*. (Informant 4)

Another security measure is caring for the women without any accompanying persons, at least in one meeting. The aim is to create a safe place without the trafficker or accompanying person monitoring the woman. Space is given to investigate if the woman is exposed to trafficking and to find out if the woman may require additional care. Obstacles to this security measure occur when the accompanying person enters the room or if the women desire company. In situations where there are language barriers, an interpreter over the telephone is used to provide the women with safety by being able to speak freely. However, obstacles to such a security measure occur if it is not possible to find an interpreter that talks the same language as the women, or if the interpreter identifies the women.

*When no interpreter is talking the same language as the women, it is challenging, of course*. (Informant 5)

Additionally, a security measure that provides both the women and the healthcare workers with safety from potential threats from accompanying persons, is that some departments in women’s healthcare are locked. That creates an awareness that unauthorized persons cannot enter the department, allowing safer care for the women. The healthcare workers can thereby protect the women and themselves from immediate danger.

*The accompanying person can be denied entry, and we can call the security guards. If they need support, the police are contacted. We do so to protect ourselves from direct violence*. (Informant 3)

### Value of confidence building

Confidence was described as an essential aspect of providing safety. The possibility of identifying women subjected to human trafficking for sexual exploitation increases if the women feel safe within the hospital. Women seek care for intimate issues, which can be perceived as an exposed situation, leaving the women reliant on the healthcare providers. The women are asked about violence and sexual exposure, the healthcare providers thereby demonstrating that it is a safe place to raise such issues. Talking in a way that the women understand is central to building confidence and providing safety. The findings also stress the importance of accepting the woman’s decisions, and not showing the woman if her decision, or her lack of action, is distressing for the healthcare providers.

*We as staff cannot get angry with the patient, we do not live their life, and we may have a completely different starting point for what would be appropriate. We can offer care, but we cannot force, and we should not blame, but it is not easy*. (Informant 2)

Another aspect of building confidence and creating safety is to provide the women with additional care and check-ups after sexual exposure. That gives an overall picture of the woman and her further care needs. It may also create a feeling of safety for the women and for the healthcare providers.

### The importance of collaborating

Regarding the identification and care of women subjected to human trafficking for sexual exploitation, the healthcare providers’ narratives emphasize the importance of collaboration both within women’s healthcare and with external authorities with other expertise. A working collaboration structure and a clear division of responsibility are essential. Also, in transferring information about the women within the collaboration it is crucial to maintain confidentiality.

#### Organizational coordination

Organizational coordination is essential in identifying and caring for a woman subjected to human trafficking for sexual exploitation. All professionals in the healthcare department must be on the same path, and the findings of this study indicate a close collaboration between the different professions within women’s healthcare most of the time. When caring for women subjected to human trafficking for sexual exploitation, the professionals gather around the woman and aim to facilitate her stay at the hospital, where she is, for the time being, in safety.

*It was such a particular case. We took care of her here so she did not have to find another place to go.* (Informant 5)

Obstacles to the organizational coordination aimed at identifying and caring for women subjected to human trafficking for sexual exploitation, are stressed in the health providers’ narratives. Time and expertise within the working group are lacking. There is a perception that patients subjected to human trafficking for sexual exploitation leave the hospital unidentified, due to a lack of understanding among the healthcare providers.

*Over the years, there are probably a lot of patients that have been here that we do not know anything about that could have been subjected to human trafficking, but we did not notice anything*. (Informant 7)

The findings highlight a desire for institutional assistance to develop a specific department for the patient population in question, offering the women time, safety, and understanding. Additionally, the findings underline the desire for institutional assistance in collecting risk factors of human trafficking for sexual exploitation.

#### Essential external network

The police, social services, and women’s emergency services are foregrounded as essential in the external network. Our findings demonstrate that the police sometimes assist women subjected to human trafficking for sexual exploitation to enter women’s healthcare. The police may then provide information about the woman to relieve her from having to repeat what she has told them about her exposure to sexual exploitation. However, the narratives underline an uncertainty regarding when to contact the police. The care of the women is the main priority for the healthcare providers, and the women are not forced to file a police report. If a woman wants to file a police report, she is assisted in doing so, however, if she does not know how to perform it. Even so, there is an insecurity among healthcare workers regarding how to address and handle these situations.

*What should be documented, and what should we not document, when should we contact the police, and when should we not contact the police*. (Informant 4)

A further hindrance demonstrated is the social services not responding to concerns about women at the hospital, which creates mistrust of the additional caring for the women. Another aspect hampering the collaboration is the women’s concealment, which limits access to essential information when caring for them. An additional challenge is that some women fear social services and will not supply healthcare providers with information. That complicates the identification and care of a woman subjected to human trafficking for sexual exploitation.

*It is difficult to do the best for the patient when there is secrecy.* (Informant 5)

#### Information transmission

The findings stress the importance of information transmission when collaborating within women’s healthcare and with external actors. Documentation of injuries on the woman’s body is given to the police and forensics. The documentation challenges are emphasized by the informants, as it is essential to provide necessary information of good quality and ensure that the legal certificates are written correctly. Moreover, when several healthcare departments provide women with care, challenges arise regarding whether the women have received questions about being subjected to violence. Lack of information transmission between departments may risk missing critical information to identify the women as subject to human trafficking for sexual exploitation, leaving the women unidentified and without further care. This indicates a need to document signs and suspicions immediately.

*You have seen something, I did not notice that in my shift, the next person sees something, the next person does not, and then it falls between the cracks*. (Informant 3)

Another stated challenge is the information transmission between the healthcare providers and the women subjected to human trafficking for sexual exploitation. The findings highlight that women lack mobile phones and cannot communicate an address, and that some women are undocumented. That may hinder additional care and support, since ways of information transmission are missing.

*We had a woman we suspected, which I still do… We were only allowed by the woman to have contact over email, which is not allowed, but I had to overlook that and email her. Her address was unknown, and she had a phone number that she did not answer. I booked an appointment for her, but she did not show up*. (Informant 9)

## Discussion

This study aimed to describe healthcare providers’ experience of identifying and caring for women subjected to human trafficking for sexual exploitation seeking women’s healthcare. The findings of the present study demonstrate that these women have limited time within healthcare, which stresses the need for healthcare providers to act swiftly at the slightest suspicion of human trafficking for sexual exploitation. The study participants have expressed, that the women in question, are in a vulnerable position when entering the hospital. Therefore, the healthcare providers argue that, providing the women with safety is paramount. As the findings demonstrate, part of the vulnerability is related to lack of mobile phones and contact information. The healthcare providers within the study argue that it can challenges the future care for these women due to the lack of avenues for information transmission. The healthcare providers, therefore, emphasizes the importance of acting when the women are inside the hospital.

The present findings align with international research [18, 21] regarding women seeking care during exploitation, and regarding the fact that a majority of the women in question leave the hospital unidentified and unaided. However, this study contributes additional insights to previous research [18, 21], that have been given restricted space in previous research. The study result highlight that the women in question have limited time within women’s healthcare, which stresses the importance of acting immediately on suspicions of human trafficking for sexual exploitation. The study findings demonstrate that the women can be in another city or country the next day without the healthcare providers being able to reach them. Previous research [34] state that the women’s personal belongings are taken away by the trafficker, which can be explained and ascertained by the present research. As demonstrated in this study, lack of mobile phones and contact information hampers additional care, e.g., if the healthcare information must go through the trafficker. Once more, the study findings highlight the importance of being attentive and acting without delay when the women are inside the hospital walls.

Other researchers [35] stress that traffickers often monitor the women inside the emergency room, and similar findings are shown in the present study. However, the present study adds to previous studies [35] by contributing suggestions for how healthcare providers can operate in such situations. The study participants, highlight the possibility of hospitalizing women for non-medical reasons when suspecting human trafficking for sexual exploitation. The study participants argued that hospitalizing women for non-medical reasons can be seen as a security measure, and expands the womens limited time within the healthcare. That enables additional care, and a possibility of providing safety by separating the women from the traffickers. However, the authors have taken into consideration that the study result may involve ethical consideration. For example, whether is ethical right to hospitalizing women without any medical reasons if there is a lack of hospital beds and other patients need medical treatments. Similarly, the study results may also require ethical consideration regarding the women’s limited time within the hospital. For example, the results show that women subjected to human trafficking for sexual exploitation may leave the hospital due to the long waiting time to see the doctor. That could, raise the question of whether that patient should be seen by the doctor before another patient who has more time but may have a more severe medical condition.

This study contributes empirical knowledge, highlighting the importance of identifying if people are subjected to human trafficking for sexual exploitation. In alignment with previous research [15], the present study has demonstrated that healthcare providers have a unique position in identifying and caring for patients subjected to human trafficking for sexual exploitation. Previous research [24] and the present results show similarities in medical signs between sexual violence and human trafficking for sexual exploitation. However, patients subjected to human trafficking for sexual exploitation have exhibited a broader general vulnerability [36]. Human trafficking for sexual exploitation is a crime that needs to be combated and eliminated [37]. The perpetrators are punishable by imprisonment, but, as mentioned before, the violation will continue as long as there is a demand for paid sexual services [14]. The study findings highlights the fact that healthcare providers are witnessing a crime scene of human trafficking for sexual exploitation, which could end up fearing the perpetrators and thereby choose to close their eyes to the problem. Nevertheless, the results from the present study give the impression of a high level of civil courage among healthcare providers.

The authors of the present study believe that level of civil courage may have grown due to a change in attitudes. The study results show an awareness of human trafficking for sexual exploitation within the healthcare setting. As mentioned in the introduction, men’s violence against women was previously regarded as a private concern [1]. Today, it is considered a severe issue that endangers world health [1]. However, development and a change of attitudes take time, meaning that the engagement in eliminating human trafficking for sexual exploitation that the healthcare providers have shown in the present study, needs to be continued. As the study demonstrate, the healthcare providers need to be on the same path in order to be able to identify women subjected to human trafficking for sexual exploitation. The findings demonstrated that experiences of healthcare providers need to be collected in view of educating the new healthcare workers and thereby improve the possibility of acting immediately on human trafficking for sexual exploitation.

The authors of the present study believe that is essential to keep the Swedish context in mind, when reflecting on the study results. The region of Västra Götaland signed the European Charter for Equality of Women and Men in Local Life already in 2008 [8]. Moreover, the healthcare providers have participated in education about human trafficking for sexual exploitation and should have developed routines for identifying patients being affected by violence [7, 26]. Thus, the Swedish healthcare workers has a history of working with the issue [7, 26]. The authors believe that the strong regulations and guidelines for combating violence and preventing inequality within the country, may have strengthened the results of this study. The authors believe that the results might have turned out differently if the study had been conducted in a country with fewer regulations. However, this is not something the authors can be sure about. Furthermore, the authors are not aware of the educational level of the respondents or if they work frequently with the issue, even though they work within the Swedish healthcare system. Additionally, the authors have taken into consideration that aspects such as free healthcare for everyone in the country, resources and time, and cooperation with external actors, also might have affected the study results. Additionally, that the patient does not have to pay from their own pocket may increase the number of persons seeking healthcare. However, it might be that this information does not reach everyone and that all patients are not aware of their rights.

### Limitations

A possible study limitation is the number of participants. However, Malterud et al. [38] argue that having a small number of informants is justified if the interviews contribute rich information. A majority of healthcare providers are female, so it is not a coincidence that all our respondents identify themselves as women. Hence, experiences from male healthcare providers are lacking. We cannot say to what extent this influences our result, but it is something that could be interesting to highlight in further studies. Language barriers between healthcare providers and women subjected to human trafficking for sexual exploitation might also have influenced the interaction and the attempts to communicate. Another limitation is that the research did not include healthcare providers from the Maternity Hospital, which is thus not represented in the findings. However, the study includes a variety of departments within women’s healthcare. It is crucial to keep the Swedish context in mind when reflecting on the study results, and the fact that the results stem from a specific geographical region in Sweden may also be seen as a limitation. Data from qualitative interview studies do not aim for generalizability, however.

## Conclusion

Healthcare providers must be attentive and act immediately if suspecting that one of their female patients is subjected to human trafficking for sexual exploitation. Due to the women’s limited time within women’s healthcare, they must provide safety and take action while the woman is in the hospital. After the woman leaves the hospital, there is a risk that she will be unreachable for the healthcare providers. The security measure of hospitalizing women for non-medical reasons extends the limited time, but it may not be without negative consequences for the women subjected to human trafficking for sexual exploitation who seek medical care during the time of exploitation. Furthermore, the healthcare providers are aware that these women sometimes leave the hospital unidentified and unaided, which demonstrates the importance of collaboration and institutional assistance in improving the current state.

## Data Availability

The datasets used and/or analyzed during the current study are available from the corresponding author on reasonable request.
